# Comprehensive pan-cancer analysis identified SLC16A3 as a potential prognostic and diagnostic biomarker

**DOI:** 10.1186/s12935-025-03791-1

**Published:** 2025-04-29

**Authors:** Ping Yang, Jiayu Yin, Gongyin Zhang, Xiaofeng Li, Tongtong Chen, Wanying Zhao, Jinhai Tang, Li Lv, Xiupeng Lv

**Affiliations:** 1 Department of Radiation Oncology, The First Affiliated Hospital of Dalian Medical University, Dalian, Liaoning China; 2 Department of Pathology, The Second Affiliated Hospital of Dalian Medical University, Dalian, Liaoning China; 3 Department of Breast and Hernia Surgery, The First Affiliated Hospital of Nanchang University, Nanchang, Jiangxi China; 4 School of Basic Medicine and Public Health, Dalian Medical University, Dalian, Liaoning China

**Keywords:** SLC16A3, Pan-cancer, Prognosis, Survival, Immune infiltration

## Abstract

SLC16A3, belonging to the SLC16 gene family, is involved in the transportation of monocarboxylate. SLC16A family members play important roles in tumorigenesis, nonetheless, the specific involvement of SLC16A3 in tumor prognosis and diagnosis in human cancers remains unelucidated. This study dealt with the exploration of SLC16A3 expression in human pan-cancer and its significance regarding disease prognosis. For this investigation, the mRNA expression data of SLC16A3 were acquired from the TCGA and the GTEx datasets. The Kaplan-Meier plots, univariate Cox regression, and the ROC curve were employed for assessing the prognostic and diagnostic significance of SLC16A3 in pan-cancer. Furthermore, the cBioPortal database was used to analyze the SLC16A3 genomic alterations. Moreover, the association of the infiltration of immune cells and immune checkpoint genes with SLC16A3 was analyzed by the TIMER database. Gene Ontology and KEGG pathway analysis were employed to explore the function of SLC16A3 in pan-cancer. The resulting data demonstrated that SLC16A3 mRNA expression was overexpressed in most cancers and its protein expression was also high across diverse cancer types. Moreover, upregulated SLC16A3 expression was linked to poor OS and PFI of certain cancers. Cox regression analysis further indicated that SLC16A3 is a risk factor for patients with PAAD, CESC, LUSC, LUAD, CHOL, LGG, MESO, and OSCC. The ROC curve revealed that SLC16A3 exhibited a high accuracy (AUC > 0.9) in BRCA, CHOL, ESCA, GBM, and KIRC prediction. Moreover, the acquired data indicated that in pan-cancer, the SLC16A3 expression exhibited correlations with immune checkpoint genes and immune cells. These findings collectively suggest that SLC16A3 holds promise as a biomarker for diagnostic and prognostic purposes in pan-cancer.

**Supplementary Information:**

The online version contains supplementary material available at 10.1186/s12935-025-03791-1.

## Introduction

The incidence of cancer has seen a rapid increase in recent years as highlighted by the global cancer statistics 2020 [1]. Among the top 10 highly prevalent cancers, lung, colorectum, liver, and stomach cancers were noted to be the highest contributors to the heightened fatality rates globally. Despite dedicated efforts to advance cancer diagnosis and management strategies, the overall survival outlook remains grim and most patients are diagnosed at advanced stages [2–4]. Consequently, there is a pressing need to innovate and devise novel approaches for diagnosing and treating cancer. At present, the usage of cancer biomarkers in diagnosis has received great attention, and an increasing number of biomarkers are being identified [5].

There is currently a significant level of interest and scrutiny directed towards understanding how lactate contributes to the behavior associated with the development of tumors [6–8]. In vitro experiments have proven that lactate is a potent inhibitor of antitumor T cells, in addition, lactate also demonstrated properties suggesting its association with the inhibition of dendritic cell (DC) differentiation [9]. The latest research has indicated that lactate activates M2-like gene expression in tumor-associated macrophages (TAMs) through a process known as histone lactylation [10]. M2-type macrophages are known for their capacity to promote tumorigenesis and development [11]. SLC16A3 (also named as MCT4), appears localized in plasma membrane and nuclear membrane [42]. SLC16A3 belongs to the SLC16 gene family, this gene family encodes multiple monocarboxylic acid transporters (MCTs), MCT1-MCT4 are lactic acid transporters, main function is to transport intracellular lactate to the extracellular compartment [12]. SLC16A3 has been associated with cell proliferation, invasion, and distant metastasis as demonstrated in prior research [13–16]. Several studies have noted that SLC16A3 played a crucial role in the development of various cancers, including lung [17–18], prostate [19], colorectal [20], and pancreatic cancers [21]. Furthermore, SLC16A3 has been shown to be capable of independently predicting the prognosis of bladder cancer [22]. Despite these findings, there has been a gap in research focusing on the expression profiles of SLC16A3 across diverse cancer types. Consequently, a comprehensive study was designed to systematically explore the significance of SLC16A3 in pan-cancer scenarios.

The expression data of SLC16A3 in human pan-cancer and healthy tissues were retrieved from various databases, including TCGA, GTEx, and TIMER2.0. The prognostic and diagnostic value of SLC16A3 in pan-cancer was then evaluated. Afterward, the research focused on elucidating whether there was any remarkable association of clinicopathological characteristics with SLC16A3 expression. Furthermore, any alterations in the SLC16A3 gene were analyzed via the cBioPortal. In addition, further exploration of the biological function of SLC16A3 was performed via Gene Ontology and KEGG analyses in the context of pan-cancer. In summary, this study indicated that SLC16A3 could serve as a potential marker for assessing prognosis and diagnosis in human pan-cancer.

## Materials and methods

### Clinical data and mRNA expression collection

All clinical data and SLC16A3 mRNA expression data were downloaded from TCGA (https://www.cancer.gov/) and GTEx (https://commonfund.nih.gov/gtex) databases. The SLC16A3 expression data from TCGA and GTEx were transformed with log2 (TPM) [23].

### SLC16A3 gene expression analysis

The TIMER2.0 database [24] (http://timer.comp-genomics.org/) was used to examine the SLC16A3 mRNA expression in TCGA human cancers and adjacent healthy tissues. Additionally, further investigation of the SLC16A3 expression profiles in pan-cancers was carried out by combining TCGA and GETx datasets. Categorization of the assessed patients was carried out per the median SLC16A3 expression into high- and low-SLC16A3 expression groups. The Wilcoxon rank sum test was conducted to assess the expression difference between the two groups [25].

### SLC16A3 protein expression analysis

Two databases, the Human Protein Atlas (HPA) [26–27] (https://www.proteinatlas.org/) and the UALCAN [28] (https://ualcan.path.uab.edu/)were searched to examine the protein expression level of SLC16A3 in human healthy tissues and cancers. Furthermore, SLC16A3 protein expression was also detected by utilizing immunohistochemistry (IHC) via a human multiorgan tissue microarray (Cat# HOrgC180PG01-1, Lot No. XT22-001, Shanghai Outdo Biotechnology). Overall, 180 paraffin-embedded tissue specimens were acquired. Among them, 91 cases were malignant tumor tissues and 89 cases were adjacent healthy tissues of cancer. The anti-SLC16A3 antibody (1:200 dilution, Cat# AF5253) was obtained from Affinity Biosciences (Jiangsu Province, China). IHC results were assessed by scoring of staining intensity and area. The intensity score ranged from 0 to 3 (0, negative; 1, light yellow; 2, light brown; 3, dark brown), and the expression area ranged from 0 to 4 (0, < 5%; 1, 5–25%; 2, 26–50%; 3, 51–75%; 4, > 75%). The SLC16A3 protein expression was determined to be equal to intensity score times expression area, with scores of 1 ~ 3 designated as low expression (+), 4–6 as moderate expression (++), and 7–12 as robust expression (+++) [29–30].

### Survival significance analysis of SLC16A3

The correlation between the expression of SLC16A3 mRNA and cancer prognosis overall survival (OS) and progression free interval (PFI) was analyzed through Kaplan-Meier plots. The diagnostic value of SLC16A3 in human cancers was examined through ROC curves [31]. In this context, the percentage of patients who survive from the beginning of randomization treatment to the end of their lives is considered as the overall survival. PFI refers to the period that a patient lives with cancer without tumor progression [32].

### Genetic alteration analysis

The SLC16A3 gene alternations in TCGA pan-cancer datasets were examined using the cBioPortal database [33] (http://www.cbioportal.org/), which is a web portal for analyzing cancer genomics.

### Correlation of SLC16A3 expression with tumor immune microenvironment

The association of SLC16A3 with the immune checkpoint genes was analyzed by the “ggplot2” R package. Furthermore, the association between SLC16A3 expression and immune cells in pan-cancer was processed by the TIMER database [34] (https://cistrome.shinyapps.io/timer/).

### Relationship between SLC16A3 expression and immune subtypes of human cancer

The TISIDB database was searched to analyze how SLC16A3 expression is linked to immune subtypes in different types of cancer [35]- [36]. TISIDB, functioning as a web portal, integrates diverse data types, including high throughput screening data, genomics, and transcriptomics data. It serves as a valuable tool for analyzing the interaction of the immune system and tumors.

### Statistical analysis

Differences in expression and correlation across the high and low SLC16A3 groups were investigated using the Wilcoxon rank sum test and the Spearman rank sum test. The log-rank test was utilized for the assessment of the Kaplan-Meier curves. R version 4.2.2 software was used for statistical analysis, with a p-value < 0.05 as the threshold of statistical significance [23]. (ns, *p* ≥ 0.05; *, *p* < 0.05; **, *p* < 0.01; ***, *p* < 0.001).

## Results

### SLC16A3 expression in human cancers

**Fig. 1:**
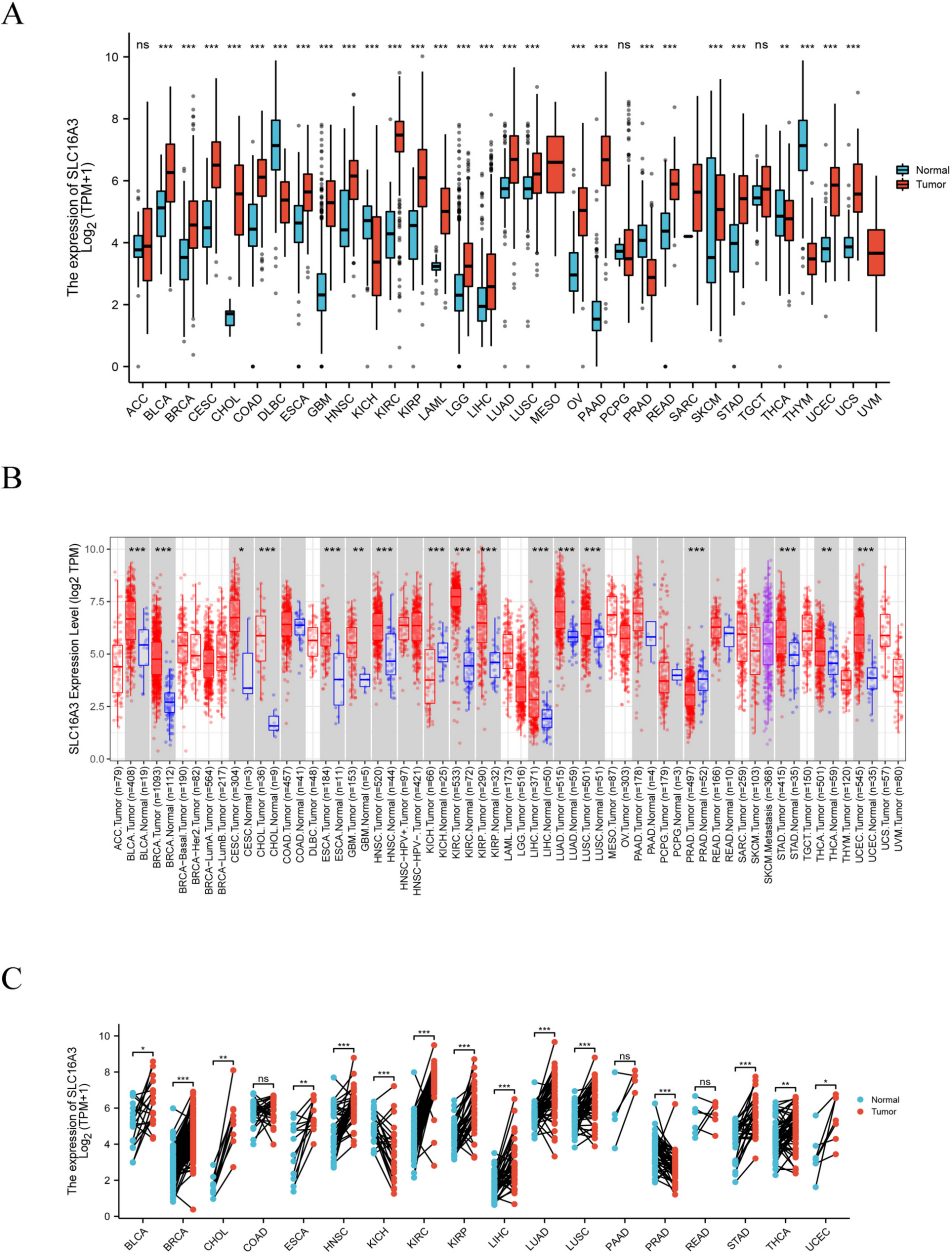
Pan-cancer analysis of SLC16A3 expression. (**A**) SLC16A3 mRNA expression in TCGA cancers and GTEx normal tissues. (**B**) SLC16A3 mRNA expression in human cancers from the TCGA database analyzed by the TIMER2.0 database. (**C**) SLC16A3 mRNA expression level in paired tumor samples based on TCGA database. (*P < 0.05, **P < 0.01, ***P < 0.001)

The SLC16A3 expression was investigated in pan-cancer and the data acquired indicated that its expression exhibited remarkable variance across diverse malignant tissues (Fig. 1A). Moreover, the results of the investigation of SLC16A3 mRNA expression in human pan-cancer using TIMER, implied that SLC16A3 exhibited remarkable upregulation in bladder urothelial carcinoma (BLCA), breast invasive carcinoma (BRCA), cervical squamous cell carcinoma and endocervical adenocarcinoma (CESC), esophageal carcinoma (ESCA), cholangiocarcinoma (CHOL), glioblastoma multiforme (GBM), head and neck squamous cell carcinoma (HNSC), kidney renal clear cell carcinoma (KIRC), kidney renal papillary cell carcinoma (KIRP), liver hepatocellular carcinoma (LIHC), lung squamous cell carcinoma (LUSC), lung adenocarcinoma (LUAD), stomach adenocarcinoma (STAD), thyroid carcinoma (THCA) and uterine corpus endometrial carcinoma UCEC compared with their adjacent healthy tissues (Fig. 1B). In addition, SLC16A3 expression in paired samples was much higher in most tumor types in comparison to healthy tissues. Nevertheless, no considerable variance was noted in the SLC16A3 expression relative to the adjacent healthy tissues of colon adenocarcinoma (COAD) and rectum adenocarcinoma (READ) (Fig. 1C).

### The protein level of SLC16A3 in human tissues

**Fig. 2:**
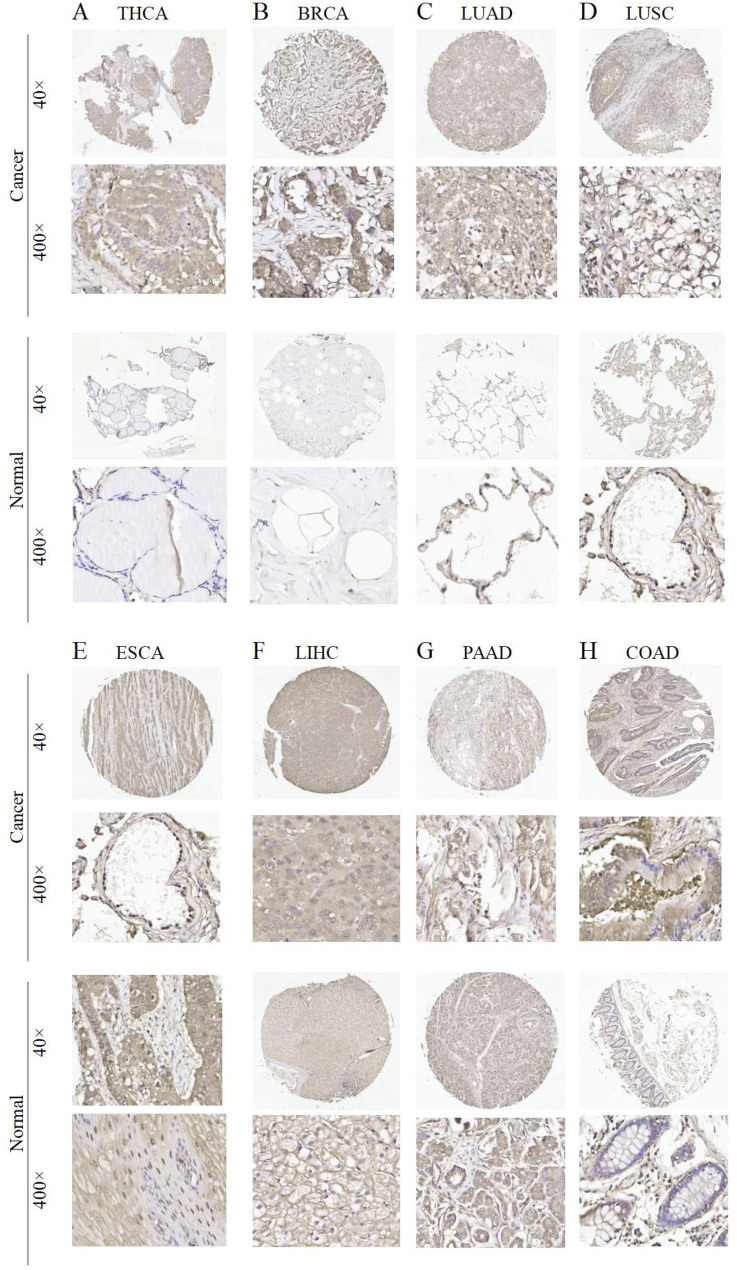
Immunohistochemistry (IHC) staining of SLC16A3 in human cancers. (**A**) THCA, (**B**) BRCA, (**C**) LUAD, (**D**) LUSC, (**E**) ESCA, (**F**) LIHC, (**G**) PAAD, and (**H**) COAD. Representative images of SLC16A3 expression in pan-cancer tissues are shown. Original magnification, ×40 and ×400

**Fig. 3:**
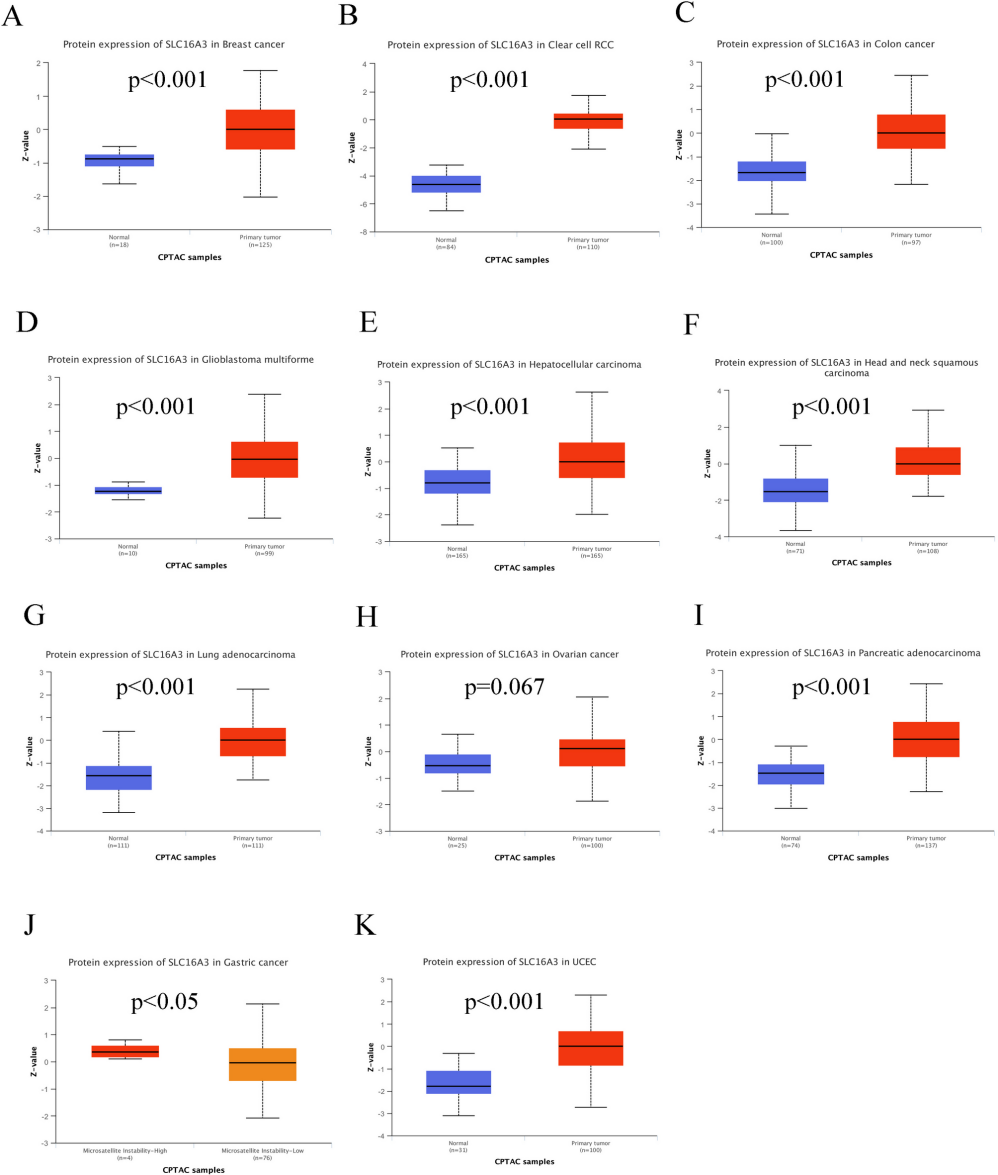
SLC16A3 protein expression analysis

Protein is the main molecule related to diseases, and changes at the level of a protein are directly related to many diseases. Thus, further investigation into the expression differences of SLC16A3 protein in tumor and normal tissues was considered necessary and was undertaken using the UALCAN and HPA databases. The outcomes of IHC, as reported in the HPA database, showed that most cancer tissues show moderate to strong cytoplasmic positivity (supplementary Figure S3A-F). Additionally, our IHC staining results were observed to be consistent with the HPA database (Fig. 2A and H). As shown in the UALCAN dataset, the SLC16A3 protein was highly expressed in BRCA, ccRCC, COAD, GBM, HCC, HNSC, LUAD, PAAD, and UCEC, but was lower in gastric cancer and did not significantly differ in ovarian cancer (Fig. 3A and K).

### Prognostic and diagnostic importance of SLC16A3 in pan-cancer

**Fig. 4:**
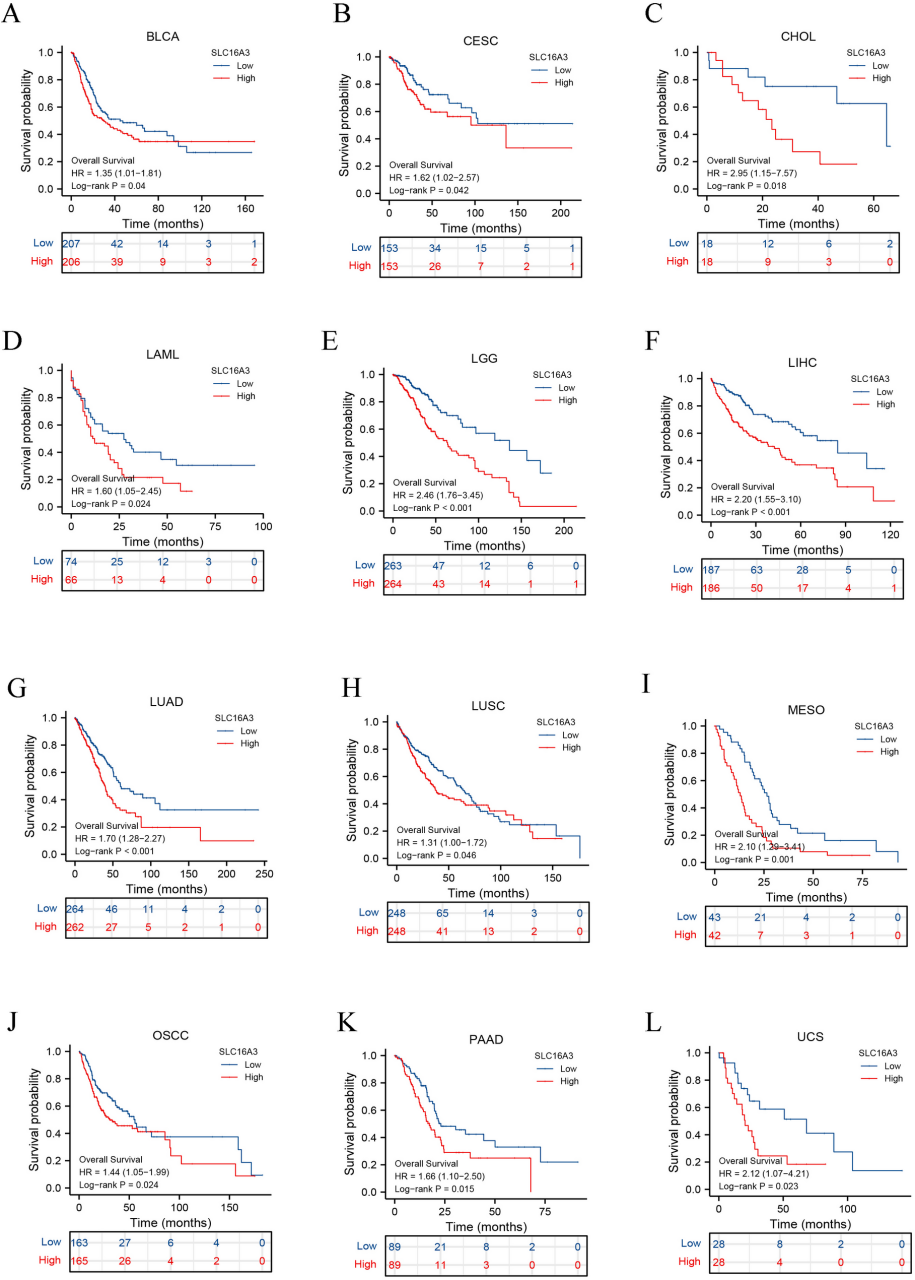
Patient overall survival analysis. (**A**-**L**) Kaplan–Meier analysis of the association between SLC16A3 expression and OS in muti-tumor types from TCGA database

**Fig. 5:**
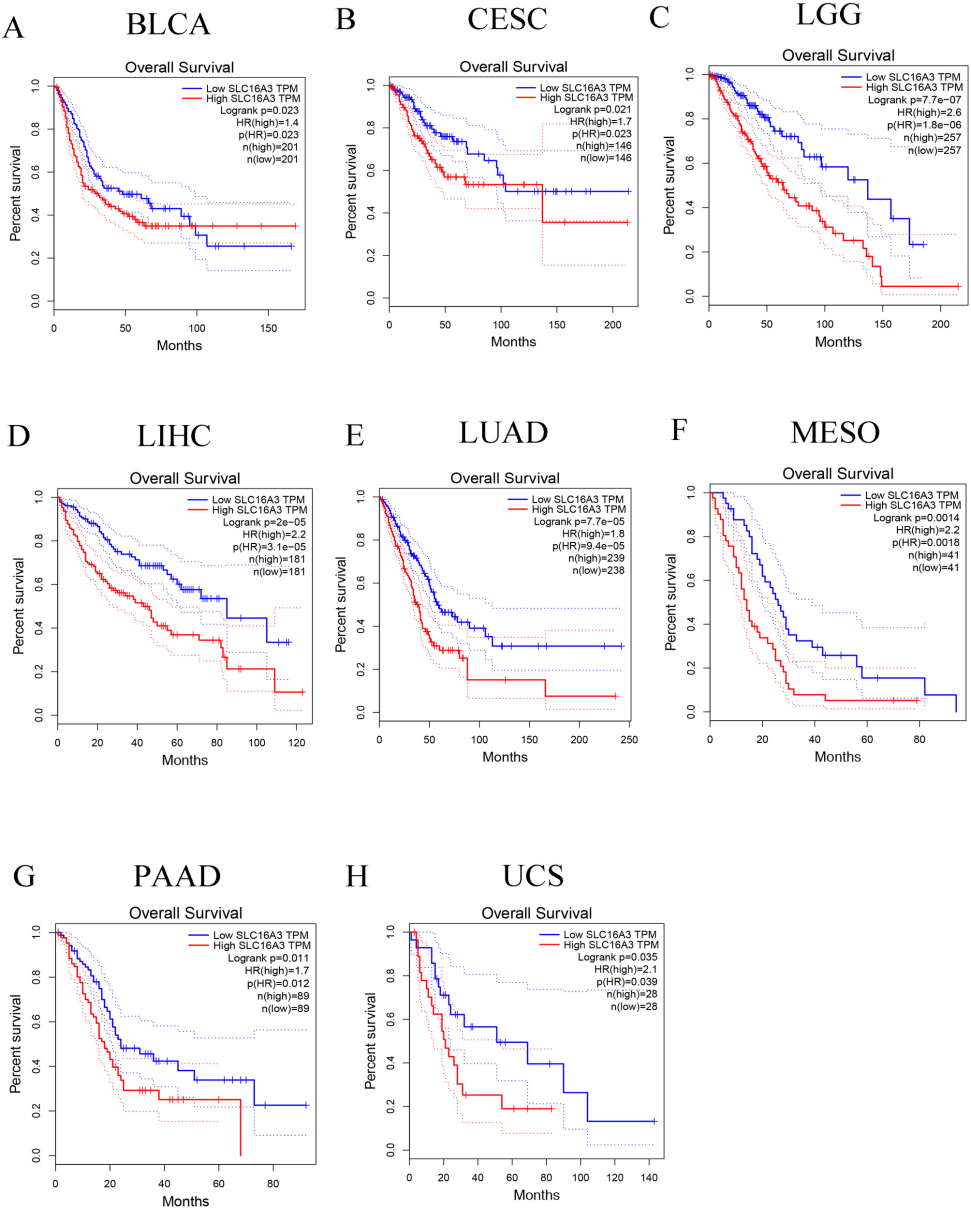
Kaplan-Meier overall survival curves of SLC16A3 in human cancers based on the GEPIA database. The median value of SLC16A3 is the cut-off value. (**A**) BLCA; (**B**) CESC; (**C**) LGG; (**D**) LIHC; (**E**) LUAD; (**F**) MESO; (**G**) PAAD; (**H**) UCS

**Fig. 6:**
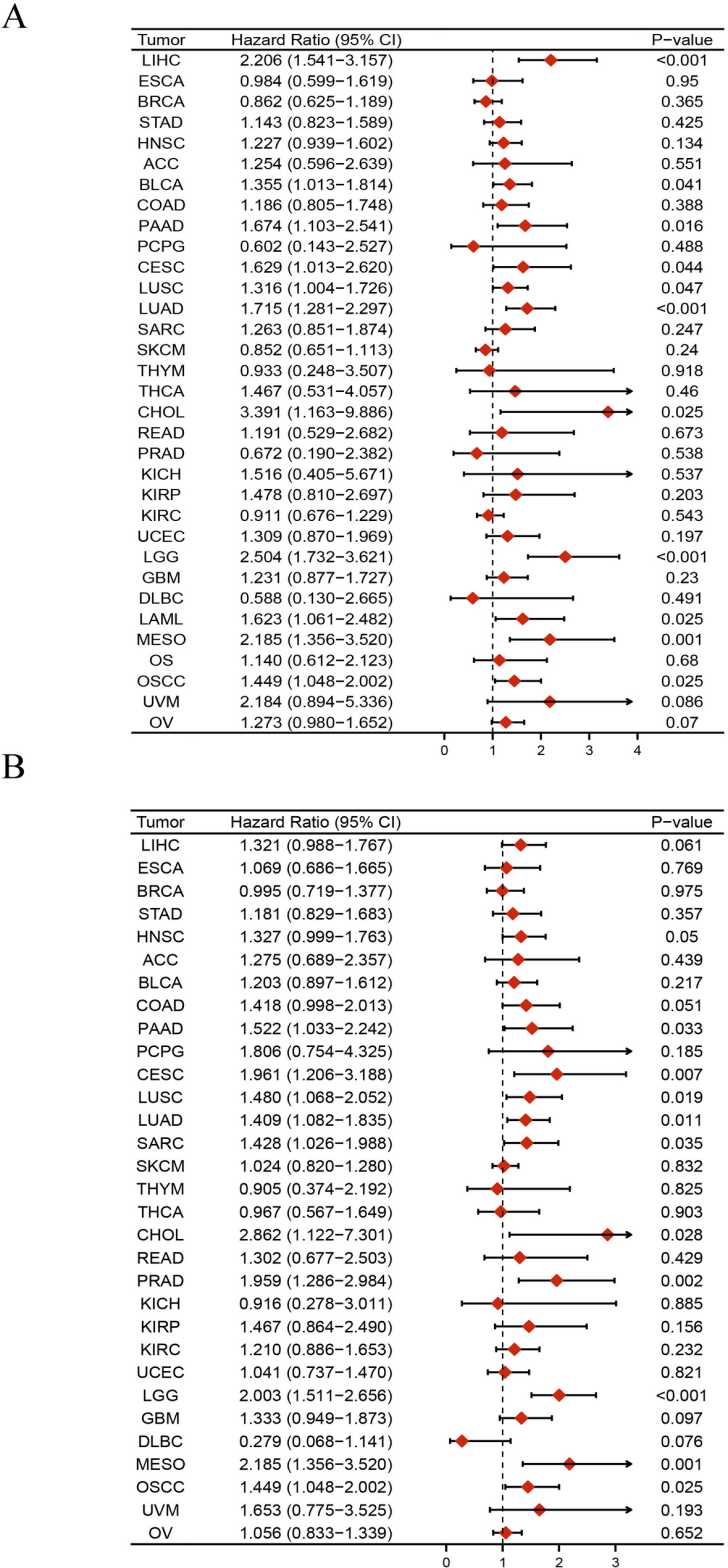
Univariate Cox regression analysis of SLC16A3. Forest map shows the univariate cox regression results of CD161 for OS (**A**) and PFI (**B**) in TCGA pan-cancer

**Fig. 7:**
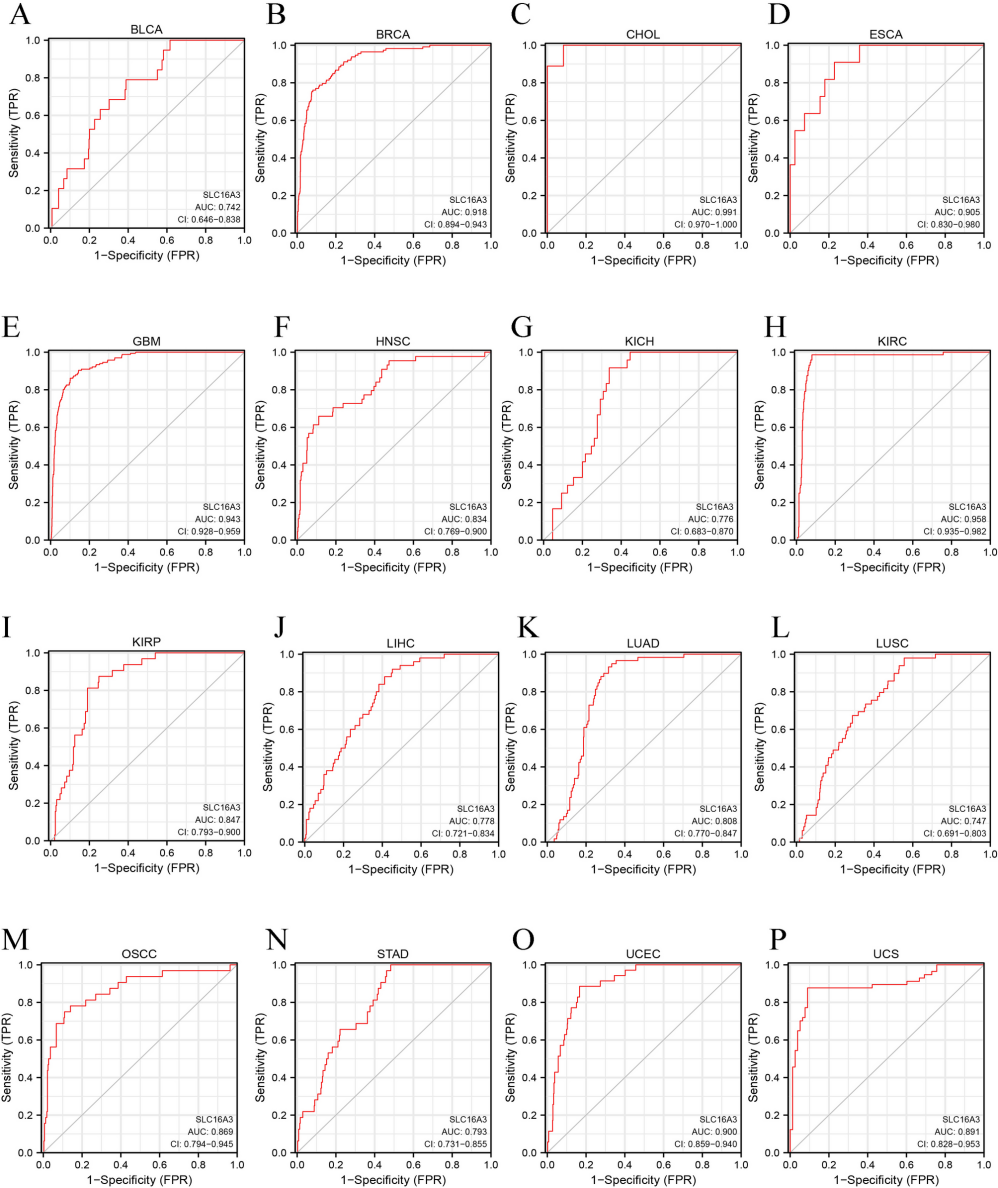
ROC curve for SLC16A3 expression in pan-cancer. (**A**) BLCA; (**B**) BRCA; (**C**) CHOL; (**D**) ESCA; (**E**) GBM; (**F**) HNSC; (**G**) KICH; (**H**) KIRC; (**I**) KIRP; (**J**) LIHC; (**K**) LUAD; (**L**) LUSC; (**M**) OSCC; (**N**) STAD; (**O**) UCEC; (**P**) UCS

The pan-cancer assessment of the prognostic and diagnostic importance of SLC16A3 demonstrated that upregulated expression of SLC16A3 and adverse OS were linked in specific cancers, encompassing BLCA, CHOL, CESC, LGG, LAML, LIHC, LUAD, LUSC, MESO, OSCC, PADD, and UCS (Fig. 4A and L). Further analysis via GEPIA, resulted in determining a robust relationship between the expression of this gene and OS in diverse cancers, encompassing BLCA, CESC, LGG, LIHC, LUAD, MESO, PADD, and UCS (Fig. 5A and H). Moreover, univariate Cox regression was utilized to confirm any potential association of SLC16A3 with the survival probability in diverse cancers. The resulting data indicated that SLC16A3 was positively correlated with the hazard ratios of OS and PFI in PAAD, CESC, LUSC, LUAD, CHOL, LGG, MESO, and OSCC. Based on the acquired data, it can be said with some degree of confidence that SLC16A3 may function as a risk factor for patients with PAAD, CESC, LUSC, LUAD, CHOL, LGG, MESO, and OSCC (Fig. 6). The ROC curve was utilized for evaluating the diagnostic potential of SLC16A3 in pan-cancer, and based on the resulting data, SLC16A3 demonstrated accuracy (AUC > 0.7) in predicting 16 types of cancer, including BLCA (AUC = 0.742), BRCA (AUC = 0.918), CHOL (AUC = 0.991), ESCA (AUC = 0.905), GBM (AUC = 0.943), HNSC (AUC = 0.834), KICH (AUC = 0.776), KIRC (AUC = 0.958), KIRP (AUC = 0.847), LIHC (AUC = 0.778), LUAD (AUC = 0.808), LUSC (AUC = 0.747), OSCC (AUC = 0.869), STAD (AUC = 0.793), UCEC (AUC = 0.900), and UCS (AUC = 0.891) (Fig. 7A and P). According to these results, SLC16A3 expression was noted to be strongly linked to the prognosis of diverse types of cancer, moreover, SLC16A3 exhibited remarkable accuracy (AUC > 0.9) in the prediction of BRCA, CHOL, ESCA, GBM, and KIRC.

### Analysis of the relationship between SLC16A3 expression and clinicopathologic characteristics

Further study was undertaken to investigate the relationship between SLC16A3 expression and the progression of clinicopathological features of the patients. In LIHC, LUAD, OSCC, and PAAD, the expression of SLC16A3 was linked to the tumor stage (pathologic stage and clinical stage). Moreover, LGG, PAAD, and PRAD also showed that SLC16A3 was associated with primary therapy treatment response. Additionally, significant associations between SLC16A3 overexpression and histologic grade were noted in OSCC, PAAD, and UCEC. According to these results, SLC16A3 expression may affect PAAD prognosis (Supplementary Figure S4).

### SLC16A3 genetic alternation analysis in pan-cancer

**Fig. 8:**
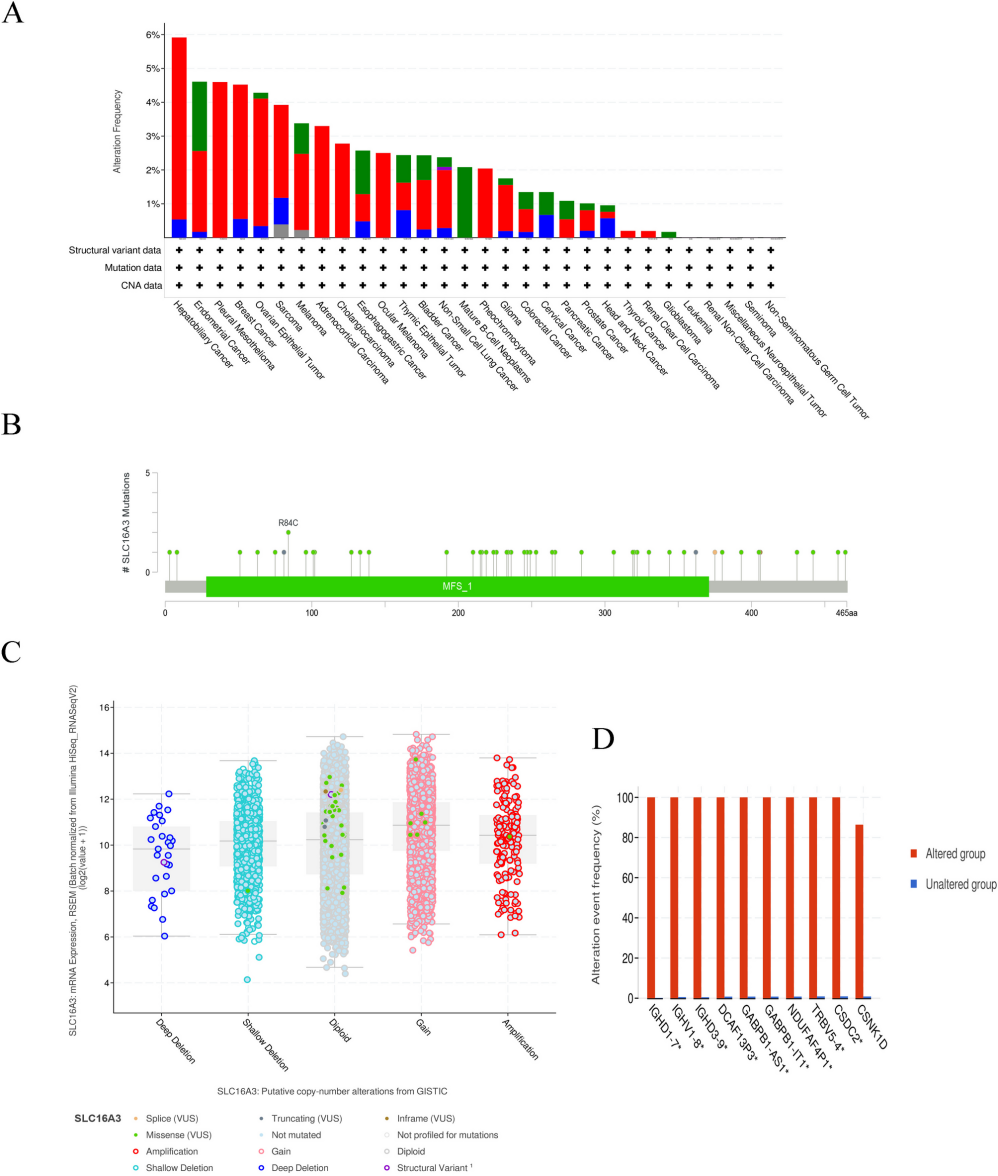
Genetic Alteration Analysis. (**A**) Genetic Alteration frequency of SLC16A3 in human pan-cancer. (**B**) The mutation types, number, and sites of the SLC16A3 genetic alterations. (**C**) The copy-number alterations of SLC16A3 in pan-cancer. (**D**) Frequency of related-gene alterations in SLC16A3-altered and unaltered groups

It was found that the SLC16A3 gene alteration rate is highest (> 4%) in hepatobiliary cancer, endometrial cancer, pleural mesothelioma, breast cancer, and ovarian epithelial tumor (Fig. 8A), Amplification, miss mutation, and deep deletion are the main.

type of frequent genetic alterations of SLC16A3. The mutation types, numbers, and sites of the SLC16A3 genetic alterations are displayed in Fig. 8B. The acquired data implied that SLC16A3 missense mutation was the major type of genetic alteration, with a total of 44 cases detected [37]. Additionally, the TCGA tumor samples exhibited two truncating mutations, one inframe mutation, one splicing mutation, and two fusion mutations (Supplementary Table 1). The most frequent copy-number variations of SLC16A3 were gain function and diploid (Fig. 8C). The altered group had a higher prevalence of gene alterations than the unaltered group did for the following genes: IGHD1-7, IGHV1-8, IGHD3-9, DCAF13P3, GABPB1-AS1, GABPB1-IT1, NDUFAF4P1, TRBV5-4, CSDC2, and CSNK1D (Fig. 8D).

### SLC16A3-related gene enrichment analysis

To investigate how SLC16A3 contributes to tumorigenesis, the GEPIA database (http://gepia.cancer-pku.cn/) was searched to obtain the top 100 genes from all tumor types that exhibited expression patterns similar to SLC16A3 (Supplementary Table 2). The KEGG pathway analysis showed that SLC16A3 might participate in oncogenesis via the “HIF − 1 signaling pathway”, “Glycolysis/Gluconeogenesis”, and “PI3K − Akt signaling pathway” (Supplementary Figure S5A). The GO term enrichment analysis [38–39] exhibited that these SLC16A3-related genes were mainly related to “response to oxygen levels”, “response to hypoxia”, and “organic acid biosynthetic process” biological processes (Supplementary Figure S5B).

### SLC16A3 expression and tumor immune microenvironment analysis

**Fig. 9:**
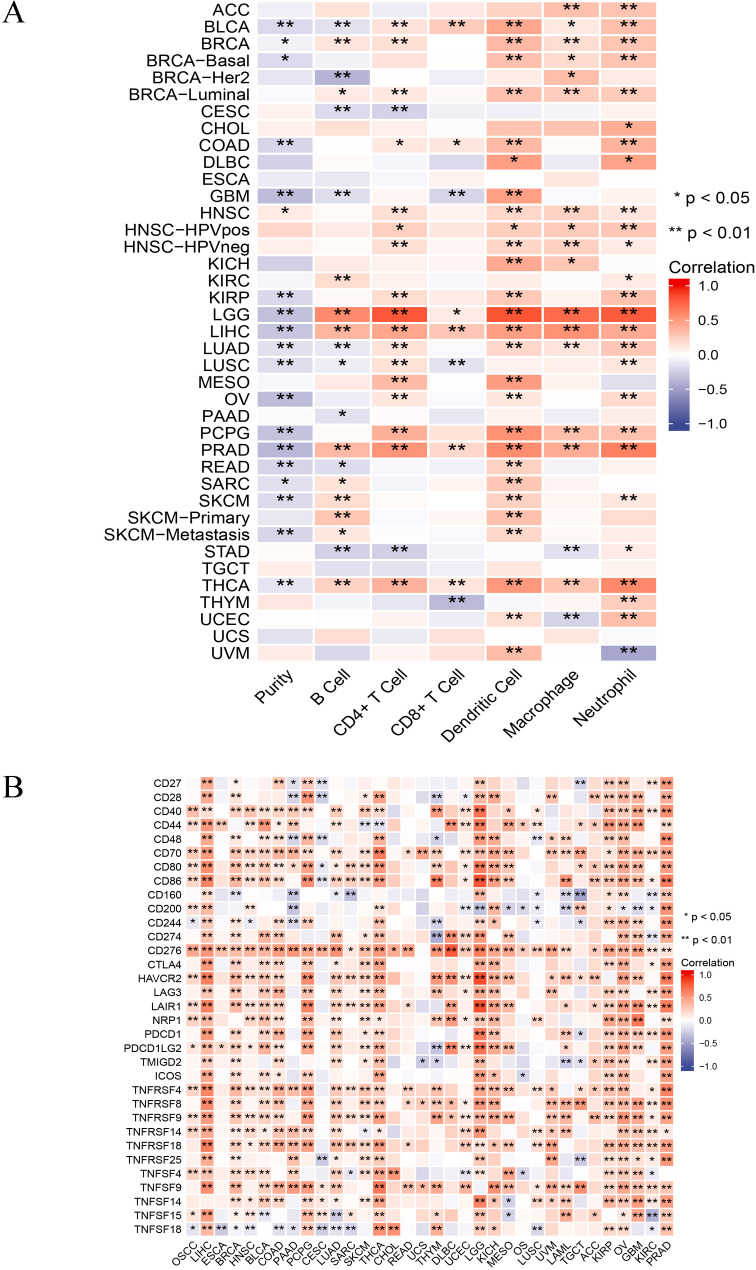
SLC16A3 expression and tumor immune microenvironment analysis. Correlation of SLC16A3 expression with immune cells (**A**) and immune checkpoint genes (**B**)

Utilizing the TIMER database, an initial exploration was conducted into the relationship between SLC16A3 expression and the infiltration of immune cells. It was observed that neutrophils, CD4 + T cells, dendritic cells, and macrophages exhibited a significant positive correlation with SLC16A3 in most tumors (Fig. 9A), as detailed in Supplementary Table 3. Moreover, evidence indicates a crucial involvement of immune checkpoint genes in immunotherapy. Consequently, further examination was carried out to investigate the correlation between SLC16A3 and ICP genes. The findings revealed a significant correlation between the mRNA level of SLC16A3 and a majority of ICP genes. Specifically, in OSCC, LIHC, BRCA, HNSC, BLCA, COAD, PCPG, LUAD, SKCM, THCA, LGG, KICH, KIRP, OV, GBM, and PRAD, SLC16A3 expression was associated with more than 20 ICP genes (Fig. 9B). SLC16A3 expression demonstrated a positive correlation with ICP genes. These results suggested that SLC16A3 might mediate immune escape and could be a potential immunotherapy target.

### SLC16A3 expression is associated with immune subtypes in pan-cancer

**Fig. 10:**
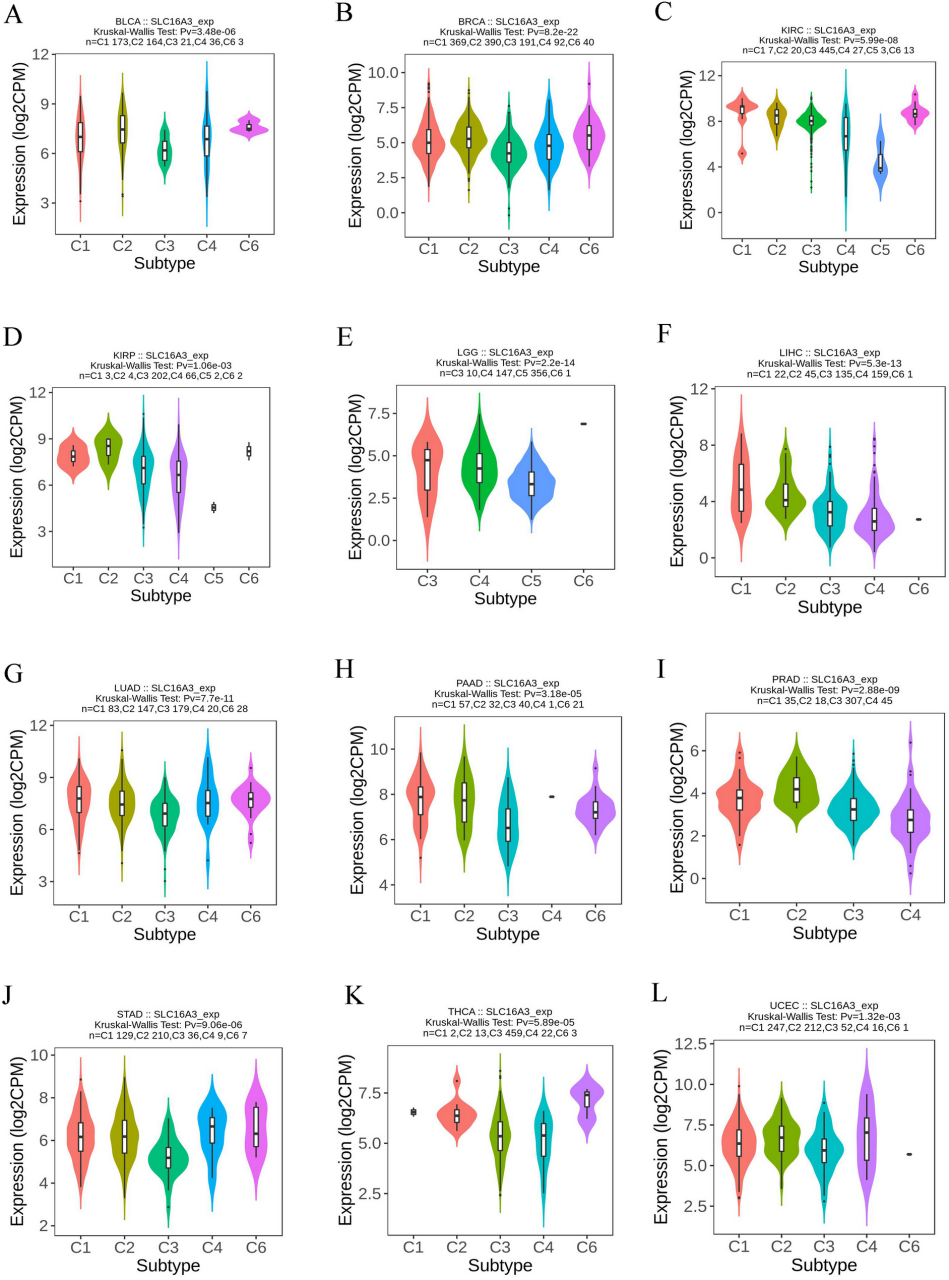
Correlation of SLC16A3 expression with pan-cancer immune subtypes. (**A**) in BLCA, (**B**) in BRCA, (**C**) in HNSC, (**D**) in KIRC, (**E**) in LGG, (**F**) in LUAD, (**G**) in LUSC, (**H**) in OV, (**I**) in PRAD, (**J**) in SKCM, (**K**) in STAD, (**L**) in UCEC

Finally, the involvement of SLC16A3 expression in the immune subtypes in human cancers was explored. Immune subtypes were classified into six types from C1 to C6 [40–41]. The study outcomes indicated a correlation between SLC16A3 and various immune subtypes in BLCA, BRCA, KIRC, KIRP, LGG, LIHC, LUAD, PAAD, PRAD, STAD, THCA, and UCEC (Fig. 10). Moreover, variations in SLC16A3 expression across different immune subtypes were observed in certain cancers. For instance, in BRCA, SLC16A3 showed upregulated expression in C2 and C6 types and downregulated expression in C3 types. From these findings, it was concluded that SLC16A3 exhibits differential expression in immune subtypes of different types of human tumors.

## Discussion

The SLC16A3 gene encodes monocarboxylate transporter4 (MCT4), predominantly located at the plasma membrane. MCT4 catalyzes the transport of monocarboxylates, including L-lactate and pyruvate, across the plasma membrane [42–43]. In past research, Warburg demonstrated that cancer cells rely on glycolysis even in the presence of oxygen [44]. Walenta et al. research reported that elevated lactate levels are associated with the incidence of metastases and poor overall survival in cancer patients [45]. Mohammed et al. have reported that hypoxia stimulates MCT4/SLC16A3 transcription, making it a HIF-1-target gene [46]. Recent studies have validated the upregulation of SLC16A3 in lung, breast, and renal cancers, suggesting its potential oncogenic role in these cancers [47–50].

Lactate is a terminal metabolite of glycolysis and SLC16A3 mediates the transport of lactate from intracellular to extracellular compartments. Lactic acid activates M2 polarization of tumour-associated macrophages (TAMs) through the process of histone lactylation, and M2-TAMs induce angiogenesis to promote tumour growth and metastasis. In addition, pathological concentrations of lactate reduce the cytotoxic activity of NK and cells [9, 51]. Li et al. [52] confirmed that SLC16A3 as an ALKBH5 target gene, SLC16A3 can reduce lactic acid concentration levels and is involved in regulating Tregs, and MDSC Accumulation in the TMEs during anti-PD-1 treatments. Another one research [53] demonstrated that the SLC16A3 inhibitor could improve the efficacy of immune checkpoint blockade. In addition, a previous study revealed that Lonidamine, as a small molecule inhibitor targeting SLC16A3, can enhance the efficacy of chemotherapy or radiotherapy [54]. Consequently, SLC16A3 it might be a possible target for cancer therapy. However, no research has yet evaluated the significance of SLC16A3 in pan-cancer. As a result, a comprehensive study was conducted to elucidate the roles of SLC16A3 in pan-cancer.

Utilizing the GeneCards dataset, it was observed that SLC16A3 mRNA is comparatively overexpressed in skeletal muscle, spleen, kidney, lung, and esophagus. Subsequently, through Immunofluorescence staining analysis from the HPA database, the subcellular localization of SLC16A3 was examined. The findings indicated a predominant presence of the SLC16A3 protein in the plasma membrane and nuclear membrane. Next, an analysis of SLC16A3 expression across 33 tumor types, in comparison to healthy tissues, was conducted using data from the TCGA and GTEx databases. The findings revealed a remarkable upregulation of SLC16A3 expression in various cancer types, including ESCA, BLCA, CESC, BRCA, CHOL, KIRC, HNSC, KIRP, LIHC, LUSC, LUAD, THCA, STAD, and UCEC. These results suggest that SLC16A3 may exert significant effects on tumor development. Furthermore, an exploration of SLC16A3 protein expression levels, conducted using the UALCAN database and immunohistochemistry data from the HPA database, revealed significant overexpression of SLC16A3 protein in ccRCC, BRCA, COAD, HNSC, HCC, GBM, LUAD, PAAD, and UCEC. Immunohistochemistry demonstrated that most cancer tissues exhibited moderate to strong cytoplasmic positivity.

Additionally, for the evaluation of the prognostic and diagnostic value of SLC16A3, analyses were conducted using the Kaplan–Meier survival curve, forest plot, and ROC curve. The Kaplan–Meier survival analysis results indicate a significant correlation between elevated SLC16A3 levels and unfavorable clinical outcomes in pan-cancer. Univariate Cox regression analysis and forest plot suggested that SLC16A3 acts as a risk factor for patients with PAAD, CESC, LUSC, LUAD, CHOL, LGG, MESO, and OSCC. The ROC curve demonstrated that SLC16A3 exhibited high accuracy (AUC > 0.9) in predicting outcomes for BRCA, CHOL, ESCA, GBM, and KIRC. These findings underscore the significant prognostic and diagnostic potential of SLC16A3 in the mentioned cancers, suggesting it could serve as a promising biomarker or therapeutic target for cancer.

The SLC16A3 gene is located on chromosome 17q25.3. Because of the SLC16A3 gene mutation, it has been extensively studied in intrahepatic cholangiocarcinoma [55]. However, there is no relevant research on SLC16A3 gene alterations in human tumors. To address this gap, a pan-cancer study of SLC16A3 gene alterations was conducted through the cBioPortal database. The investigation revealed that mutations in SLC16A3 were most prevalent in hepatobiliary cancer (> 5%), followed by endometrial cancer, pleural mesothelioma, breast cancer, and ovarian Epithelial tumor (> 4%). Notably, the co-occurrence of IGHD1-7, IGHV1-8, IGHD3-9, DCAF13P3, GABPB1-AS1, GABPB1-IT1, NDUFAF4P1, TRBV5-4, CSDC2, and CSNK1D alterations was observed within the SLC16A3 alteration group.Using GEPIA [56], a set of genes exhibiting similar expression patterns to SLC16A3 across diverse tumors was identified. KEGG pathway analysis revealed their association with the HIF − 1 signaling pathway or Glycolysis/Gluconeogenesis. GO analysis revealed that SLC16A3 plays a major role in hypoxia regulation, which was consistent with previous studies [56–59].

Previous research demonstrated that elevated levels of MCT4 (SLC16A3) can contribute to immunosuppression in hepatocellular carcinoma [61]. Therefore, this study speculates that SLC16A3 might also be involved in immunity regulation in other human tumors. Using the TIMER database, it was found that neutrophils, CD4 + T cells, dendritic cells, and macrophages exhibited a significant positive correlation with SLC16A3 in most tumors. The relationship between SLC16A3 and immune checkpoint genes was further investigated. The results demonstrated a significant association between SLC16A3 and a majority of immune checkpoint genes. These findings strongly suggested the potential of SLC16A3 as an immunotherapy target. Lastly, the investigation delved into the expression of SLC16A3 across various immune subtypes in human cancers. The outcomes revealed significant differences in SLC16A3 expression among distinct immune subtypes, implying its involvement in immune regulation and its potential as a diagnostic biomarker for human cancer.

Although our research provides strong evidence for the potential role of SLC16A3 in the prognosis prediction and immunotherapy of many cancers, there are still certain limitations to this study. Firstly, our research data comes from different databases, so there may be data bias. Secondly, since this study is mainly based on bioinformatics methods, the analysis results are mostly correlation analysis, so there is a lack of sufficient in vivo and in vitro biological experimental verification. Thirdly, although high expression of SLC16A3 is associated with poor clinical survival outcomes in cancer patients, it is still unclear how SLC16A3 affects patient clinical outcomes through the immune system and further research is needed.

## Conclusion

SLC16A3 has the potential to function as a biomarker for diagnostic and prognostic purposes in pan-cancer. Additionally, it could represent a novel target for immunotherapy.

## Electronic supplementary material

Below is the link to the electronic supplementary material.

Supplementary Material 1: 50 mutations information.

Supplementary Material 2: The top 100 SLC16A3-related genes.

Supplementary Material 3: Correlation between SLC16A3 expression and six immune cells in pan-caner.

Supplementary Material 4: The correlation between SCL16A3 expression and different clinical features in pan-cancer. (A-D) Tumor stage; (E-G) primary therapy treatment response; (H-J) histologic grade.

Supplementary Material 5: GO and KEGG gene enrichment analysis.

Supplementary Material 6: SLC16A3 localization and expression under physiological conditions.

Supplementary Material 7: Immunohistochemistry staining of SLC16A3 in human cancers from the Human Protein Atlas (HPA) database.

Supplementary Material 8: SLC16A3 localization and expression under physiological conditions. (A) SLC16A3 mRNA expressions in human normal tissues from the GTEx database. (B) the subcellular localization of SLC16A3.

Supplementary Material 9: Immunofluorescence staining of the subcellular distribution of SLC16A3.

## Data Availability

The original datasets in our study can be obtained from online repositories. The names of the repository/repositories and accession number(s) can be found in the article/Supplementary Material.
