# Supplementary figures and images for: Comprehensive pan-cancer analysis identified SLC16A3 as a potential prognostic and diagnostic biomarker
Source: Cancer Cell Int. 2025 Apr 29;25:168. doi: 10.1186/s12935-025-03791-1 (PMC12039109; doi:10.1186/s12935-025-03791-1)

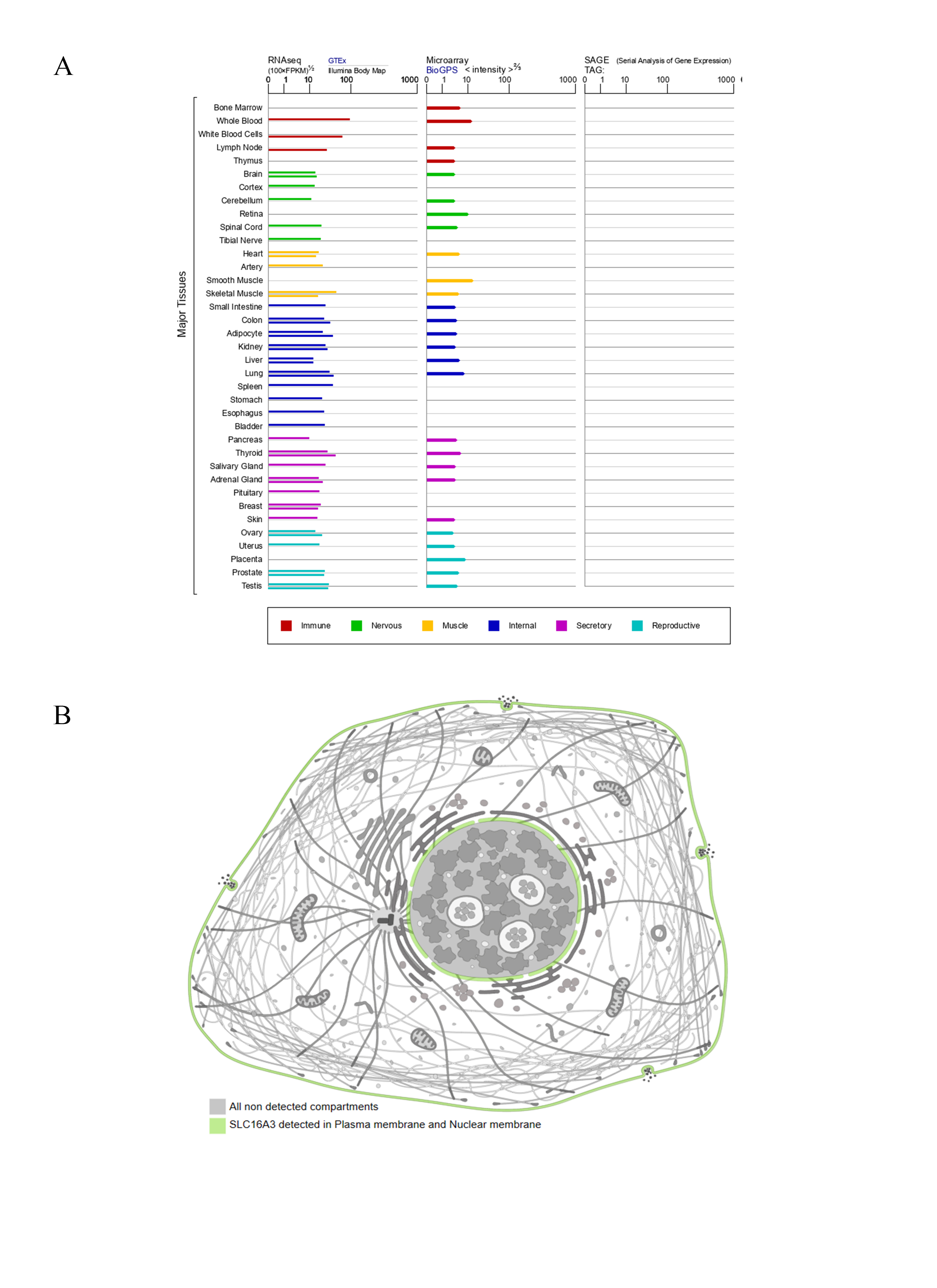

Supplement: Supplementary file 1 — Supplementary Material 1: 50 mutations information. [file 12935_2025_3791_MOESM1_ESM.jpg]

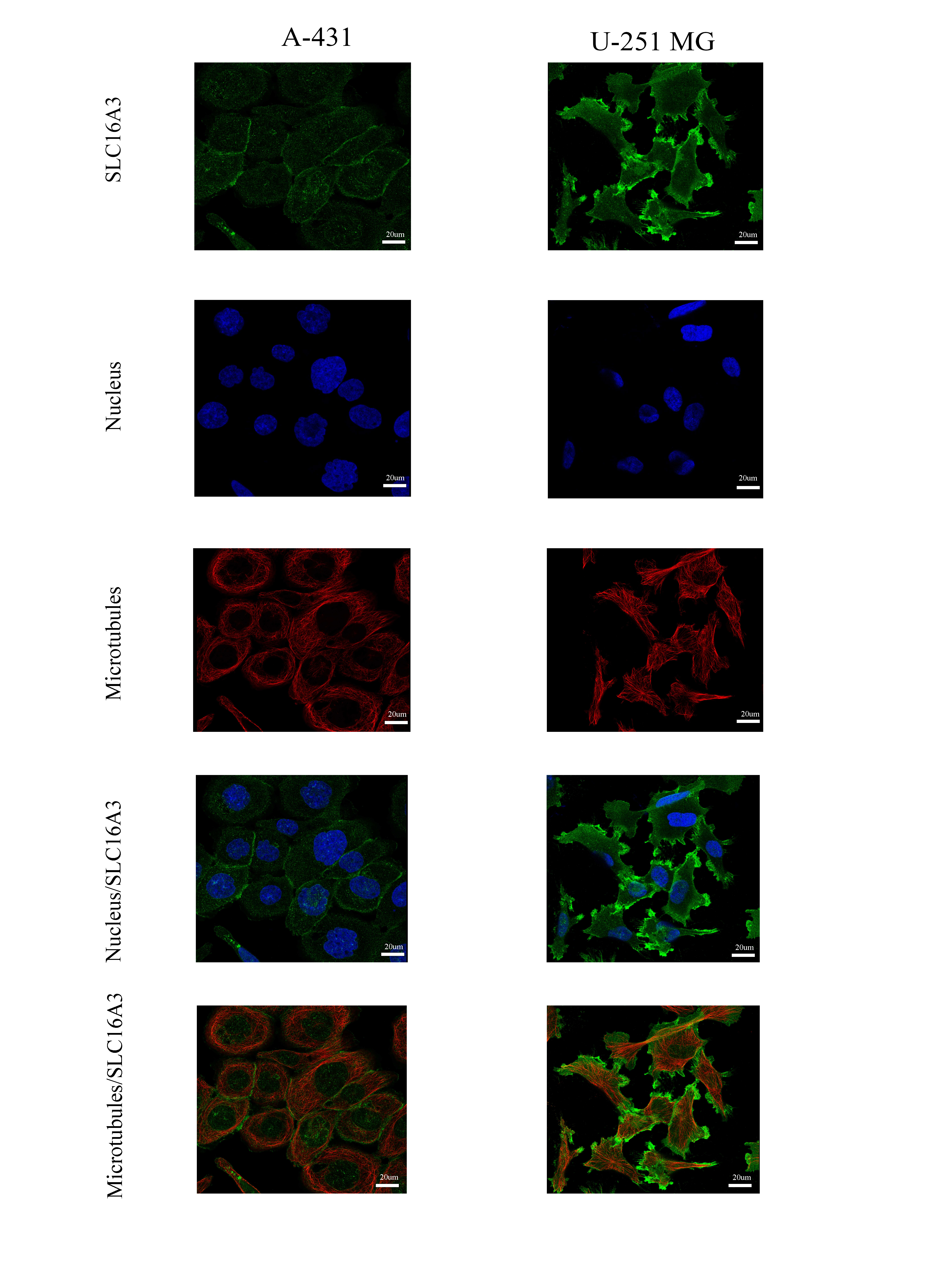

Supplement: Supplementary file 2 — Supplementary Material 2: The top 100 SLC16A3-related genes. [file 12935_2025_3791_MOESM2_ESM.jpg]

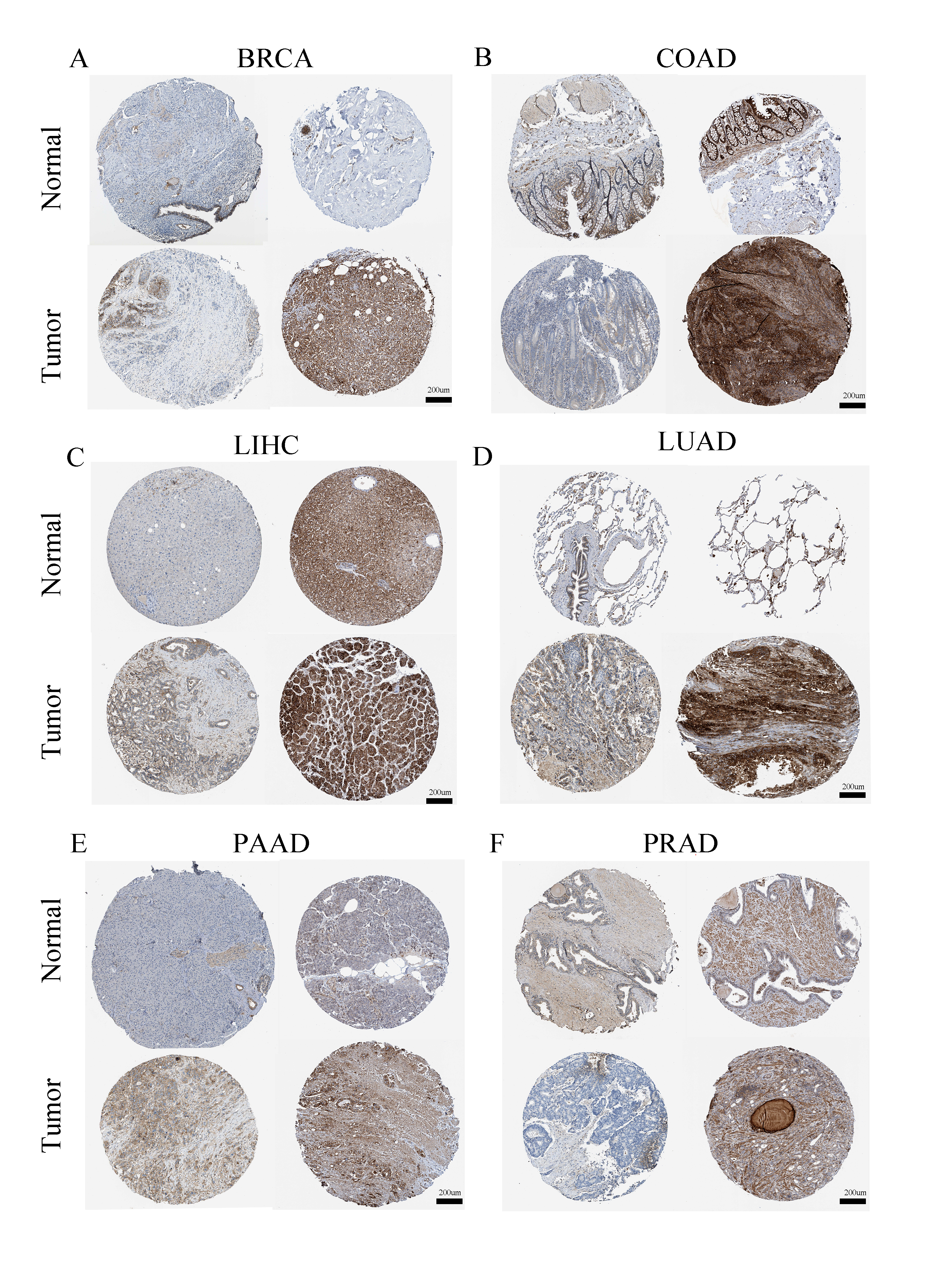

Supplement: Supplementary file 3 — Supplementary Material 3: Correlation between SLC16A3 expression and six immune cells in pan-caner. [file 12935_2025_3791_MOESM3_ESM.jpg]

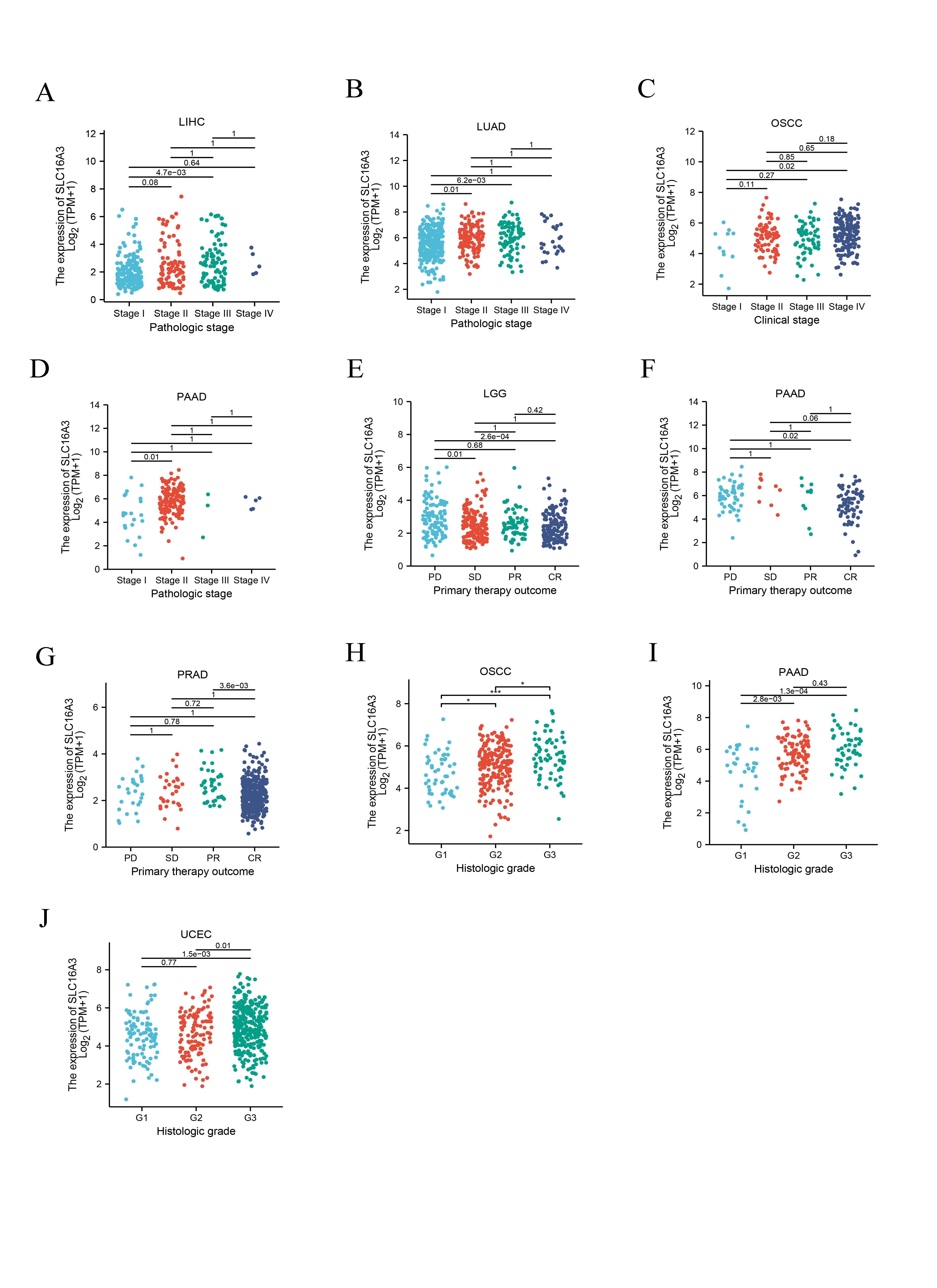

Supplement: Supplementary file 4 — Supplementary Material 4: The correlation between SCL16A3 expression and different clinical features in pan-cancer. (A-D) Tumor stage; (E-G) primary therapy treatment response; (H-J) histologic grade. [file 12935_2025_3791_MOESM4_ESM.jpg]

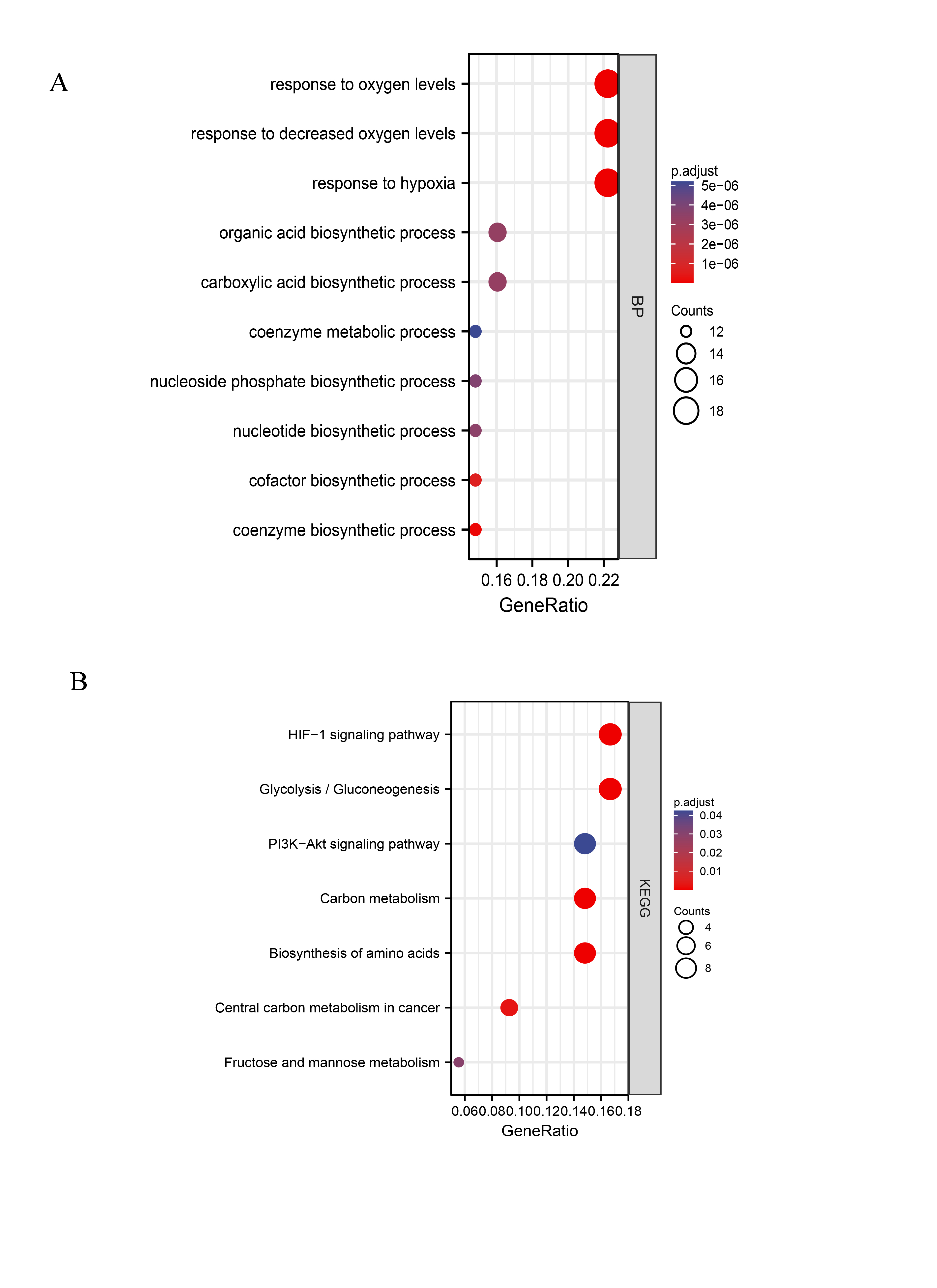

Supplement: Supplementary file 5 — Supplementary Material 5: GO and KEGG gene enrichment analysis. [file 12935_2025_3791_MOESM5_ESM.jpg]
