# Supplementary material for: Comprehensive pan-cancer analysis identified SLC16A3 as a potential prognostic and diagnostic biomarker
Source: Cancer Cell Int. 2025 Apr 29;25:168. doi: 10.1186/s12935-025-03791-1 (PMC12039109; doi:10.1186/s12935-025-03791-1)
Supplement: Supplementary file 6 — Supplementary Material 6: SLC16A3 localization and expression under physiological conditions. [file 12935_2025_3791_MOESM6_ESM.docx]

**SLC16A3 localization and expression under physiological conditions**

Based on the GeneCards dataset, we found that SLC16A3 mRNA was expressed in various human tissues. Furthermore, in some tissues, such as spleen, kidney, lung, and esophagus, these tissues have a high level of expression (Supplementary Figure S1A). Additionally, when delving into the subcellular localization of SLC16A3 using information from the HPA database, it was found that the protein primarily resides in two key cellular structures—plasma membrane and nuclear membrane (Supplementary Figure S1B). Immunofluorescence staining, adopted from the HPA database, confirmed the localization of the SLC16A3 protein. The resulting data indicated that SLC16A3 was colocalized with nuclear and plasma membrane markers in A-431 and U-251MG cells (Supplementary Figure S2).
